# A facile novel synthesis of AgCuO_2_ delafossite nanoparticles and evaluation of their antimicrobial activity

**DOI:** 10.1038/s41598-023-30255-1

**Published:** 2023-02-23

**Authors:** Ebtesam E. Ateia, M. M. Arman, Amira T. Mohamed

**Affiliations:** Physics Department, Faculty of Science, Cairo University, Giza, Egypt

**Keywords:** Nanoscale materials, Structural materials, Nanoscale materials, Design, synthesis and processing, Nanoscale biophysics

## Abstract

Bi-functional nano-oxides are of growing interest to address environmental issues. In the present study, the structural and magnetic data are presented together with the antimicrobial activities (AMA). For the first time, silver delafossite oxide (AgCuO_2_) is successfully fabricated using a simple, low-cost technique to target antibiotic photodegradation and inactivation of model waterborne pathogens. It is prepared with an equimolar initial Ag^+^:Cu^+^ concentration ratio. The structure, morphology, and magnetic properties are studied by different characterization techniques. The size and shape of AgCuO_2_ NPs, in addition to their structural polytypes of 2H (hexagonal) or 3R (rhombohedral), are dependent on the preparation conditions. The existence of Cu, Ag, and O in the synthesized delafossite AgCuO_2_ NPs with no evidence of any impurity is ratified by the XPS spectrum. AFM measurements are taken to characterize the surface morphologies of AgCuO_2_. The distributed spiks are evaluated by roughness kurtosis (Rku). The roughness kurtosis has a value of 2.65 (< 3), indicating that the prepared sample is classified as bumpy. The prepared sample has 13.0, 10.0, 14.0, and 14.0 mm Inhibition Zone Diameter (IZD) antimicrobial activity against gram-positive *Bacillus subtilis* (*B. subtilis*), *Bacillus cereus* (*B. cereus*), *Enterococcus faecalis* (*E. faecalis*), and *Staphylococcus aureus* (*S. aureus*), respectively. The IZD for gram-negative *Escherichia coli* (*E. coli*), *Neisseria Gonorrhoeae* (*N. Gonorrhoeae*), *Pseudomonas aeruginosa* (*P. aeruginosa*), and *Salmonella typhimrium* (*S. typhimrium*) were found to be 12.0, 13.0, 14.0, and 13.0 mm, respectively. Therefore, the AgCuO_2_ NPs reveal excellent antimicrobial efficiency, and they can be effortlessly separated using a tiny magnet or a simple magnetic separator. The adequate cytotoxicity and magnetic characteristics of the antimicrobial sample suggest a promising future for it in biomedical applications.

## Introduction

The importance of delafossite materials can be attributed to their characterization. They can be classified from insulators to conductors according to their conductivity (σ)^1–4^. For most Cu and Ag-based compounds, the delafossite can be classified as a semiconductor (SC). Pt and Pd-based alloys have good metallic properties^5^. Furthermore, due to its good electric and optical properties, it is classified as a transparent conducting oxide (TCO)^6–8^. The delafossite structure with a general formula contains two cations with different oxidation states (+ 1 and + 3), which provide different sites for further reaction.

Cu, Pt, Pd, or Ag are examples of typical A cations. The B cation is found in distorted edge-shared BO_6_ octahedra (B) and has a + 3 oxidation state. The cations in the B site can either be p-block metals like In, Ga, and Al or transition metal cations like Co, Fe, and Y^9–11^. The delafossite structure consists of two alternating layers: a layer of edge-sharing BO_6_ octahedra and a planar layer of A cations in a triangular pattern with respect to the c-axis^12^. Their NPs also have distinctive characteristics, such as a wide range of chemical compositions, a large surface area, and two active sites for doping with various oxidation states^13^.

In recent eras, the increase in microorganisms’ resistance to antibiotic treatments has become a concern due to its potentially harmful effects on human health. Therefore, it is critical to find non-precious alternatives based on earth-abundant elements for the control of bacterial and fungal proliferation in uncontrolled environments.

Oxides are the most stable and effective inorganic materials for antimicrobial activity (AMA)^14,15^. Cu, Ni, Fe, and Co-based non-noble transition metal oxides have received a great deal of attention to date. However, delafossite oxides have received little research to date, but they show massive promise for discovering new physics governing the interplay of itinerant and correlated electrons in solids^16^. Among various delafossites, AgCuO_2_ is particularly desirable for scientific research and potential applications. One of the remarkable and alluring features of AgCuO_2_ is its appropriate antibacterial efficiency.

Various approaches, mainly solid state^17^, sol–gel^18^, co-precipitation^19^, and hydrothermal precursor^20^, have been utilized to synthesize delafossites, but to the best of our knowledge, no prior studies have been conducted using the citrate-auto combustion method. Interestingly, the architecture adopted by the various ABO_2_ compounds actually depends on the type of cations and the preparation technique. In this regard, the aim of the present work is to synthesize AgCuO_2_ NPs with an easy and low-cost technique. The specific objective is to investigate the effect of the preparation method on the size, shape, and polytype of AgCuO_2_ NPs.

The structure and morphology analyses were carried out via X-ray powder diffraction (XRD) and XPS. The morphology of the AgCuO_2_ was studied using HRTEM, FESEM, and AFM. The magnetic properties of AgCuO_2_ NPs were studied using VSM. The authors aimed to examine the AMA of the investigated sample.

## Experimental techniques

### Preparation of AgCuO_2_ nanoparticles

**Figure 1:**
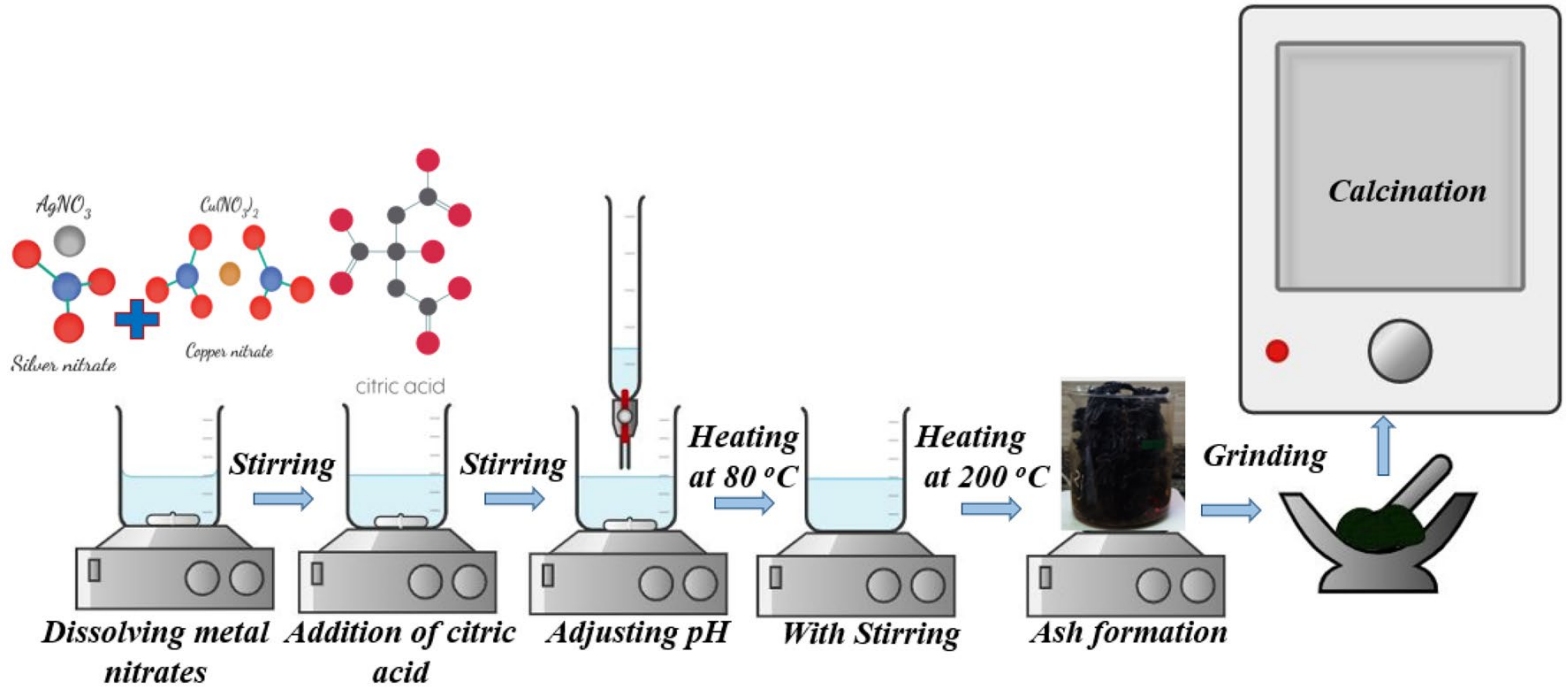
Schematic illustration for the preparation of AgCuO_2_.

The sample AgCuO_2_ was synthesized using the citrate combustion method^21,22^. As shown in Fig. 1, the stoichiometric ratios of high purity Ag (NO_3_), Cu (NO_3_)_2_⋅3H_2_O, and citric acid were dissolved in distilled water. The synthesized sample was heated at 400 °C for 4 h at a heating/cooling rate of 4 °C/min.

### Sample analysis and characterizations

X-ray powder diffraction (XRD) was performed using a Proker D8 advanced X-ray diffractometer with CuK radiation (λ = 1.5418 Å). The morphology of the investigated sample was studied using Field Emission Scanning Electron Microscopy (FESEM), Atomic Force Microscopy (AFM) and High-Resolution Transmission Electron Microscopy (HRTEM). An EDAX Genesis instrument connected to the SEM column was used to conduct energy-dispersive spectroscopy (EDAX) analysis. SEM Model Quanta 250 FEG is attached. The sample's X-ray photoelectron spectra (XPS) were recorded using K-ALPHA (Thermal Fisher Scientific, USA) with monochromatic X-ray Al K-alpha radiation. Using CASAXPS software, all XPS spectral peaks were fitted, and the data analysis included Shirley background subtraction and spectra normalization. The magnetic hysteresis loop was performed utilizing a vibrating sample magnetometer (VSM; EG & G Model No; 1551, USA) with a maximum applied field of 20 kOe at room temperature.

### Antimicrobial activity test

A modified Kirby-Bauer disc diffusion method^23^ was used to determine the antimicrobial activity (AMA) of the tested sample. The AMA was tested in vitro using nutritional agar medium against gram-positive bacteria such as *Bacillus subtilis *(*B. subtilis*),* Bacillus cereus *(*B. cereus*), *Enterococcus faecalis* (*E. faecalis*), *Staphylococcus aureus *(*S. aureus*), and gram-negative bacteria such as *Candida albicans* (*C. albicans*) *and Aspergillus*. The sterilized media were added to each of the sterilized Petri dishes (20–25 mL), and they were left to harden at 30 °C. A 1.5 × 10^5^ CFU mL^−1^ microbial suspension was created in sterile saline. 100 μL of the tested compound's solution were added to each well, using a micropipette. In order to test for AMA, the samples were incubated at 37 °C for 24 h, it results in the formation of a zone of inhibition in the form of microbial growth retardation. The zones of inhibition in this experiment were measured on a millimeter scale.

## Results and discussion

### Structural analyses

**Figure 2:**
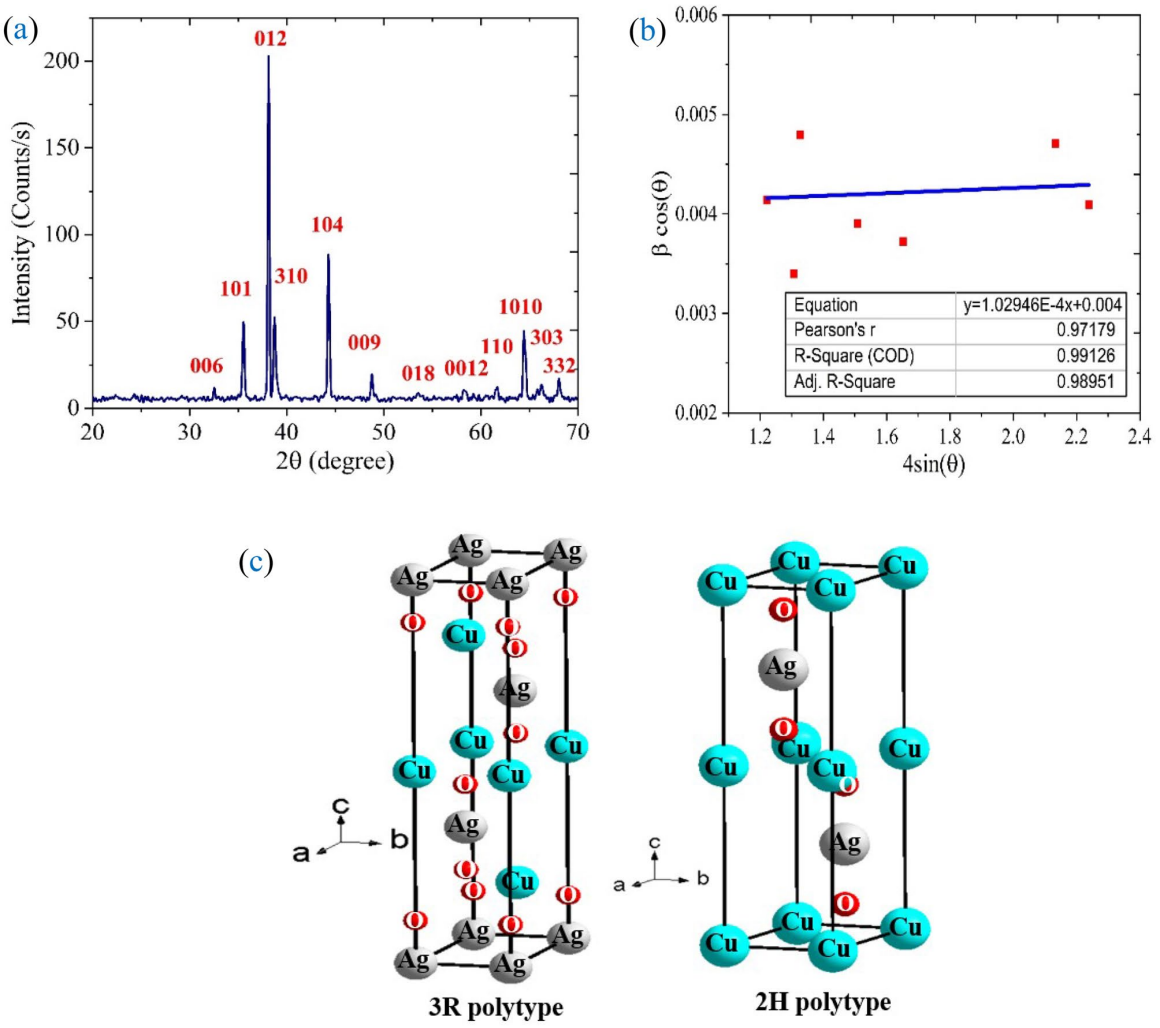
(**a**) XRD pattern of AgCuO_2_ sample, (**b**) Williamson-Hall plot, and (**c**) 3R and 2H Polytypes of AgCuO_2_.

ABO_2_'s structure is based on alternate layers of monovalent cations linearly coordinated with oxygen anions and slightly distorted edge-shared BO_6_ octahedra. The delafossite-type oxides can crystallize into the rhombohedral system 3R or the hexagonal crystal system 2H, depending on how these layers are arranged. For the 3R polytype and the 2H polytype, the layers in the delafossite structure are oriented along the c-axis in the following sequences: AaBbCcAa and AaBbAa, respectively. Capital and small letters signify alternate layers of monovalent A^+^ cations and B^3+^O_6_ octahedrons, respectively. In another way, the identical atom locations are repeated every third layer in a 3R polytype and every second layer in a 2H polytype (Fig. 2c).

Figure 2a shows the XRD diffraction pattern for AgCuO_2_ NPs. It is clear from the figure that most of the diffraction peaks (006, 101, 012, 310, 104, 009, 018, 0012, and 1010) can be indexed as 3R-AgCuO_2_ rhombohedral, R-3m, and very little 2H-AgCuO_2_ (hexagonal, P63/mmc). The diffraction peaks are noticed nearly at the diffraction angles of 38.09°, 44.3° and 64. 9°, and are labelled as (012), (104) and (1010), compared with the Ag XRD pattern. The detected peaks ratified the presence of silver NPs in the investigated system.

These facts can be rationalized by comparing and interpreting the respective crystal structures based on the partial structures of the metal atoms. These are equivalent and fit an ordered variant of AgIAgIIICuICuIIO_2_ with diverse orientations of the AgO_4_ squares and AgO_2_ dumbbells in AgCuO_2_ (see Fig. 6) as relevant from XPS (as will be discussed later).

Furthermore, both crystallite size “D” and lattice strain “ε” have a great effect on peak broadening. As a result, the Williamson-Hall (W–H) method is used for the examination^24^. AgCuO_2_ has a relatively low ε, as evidenced by the intercept and slope of the (W–H) curve in Fig. 2b, which indicates D to be 36.23 nm and ε to be 1.02910^–4^.

**Figure 3:**
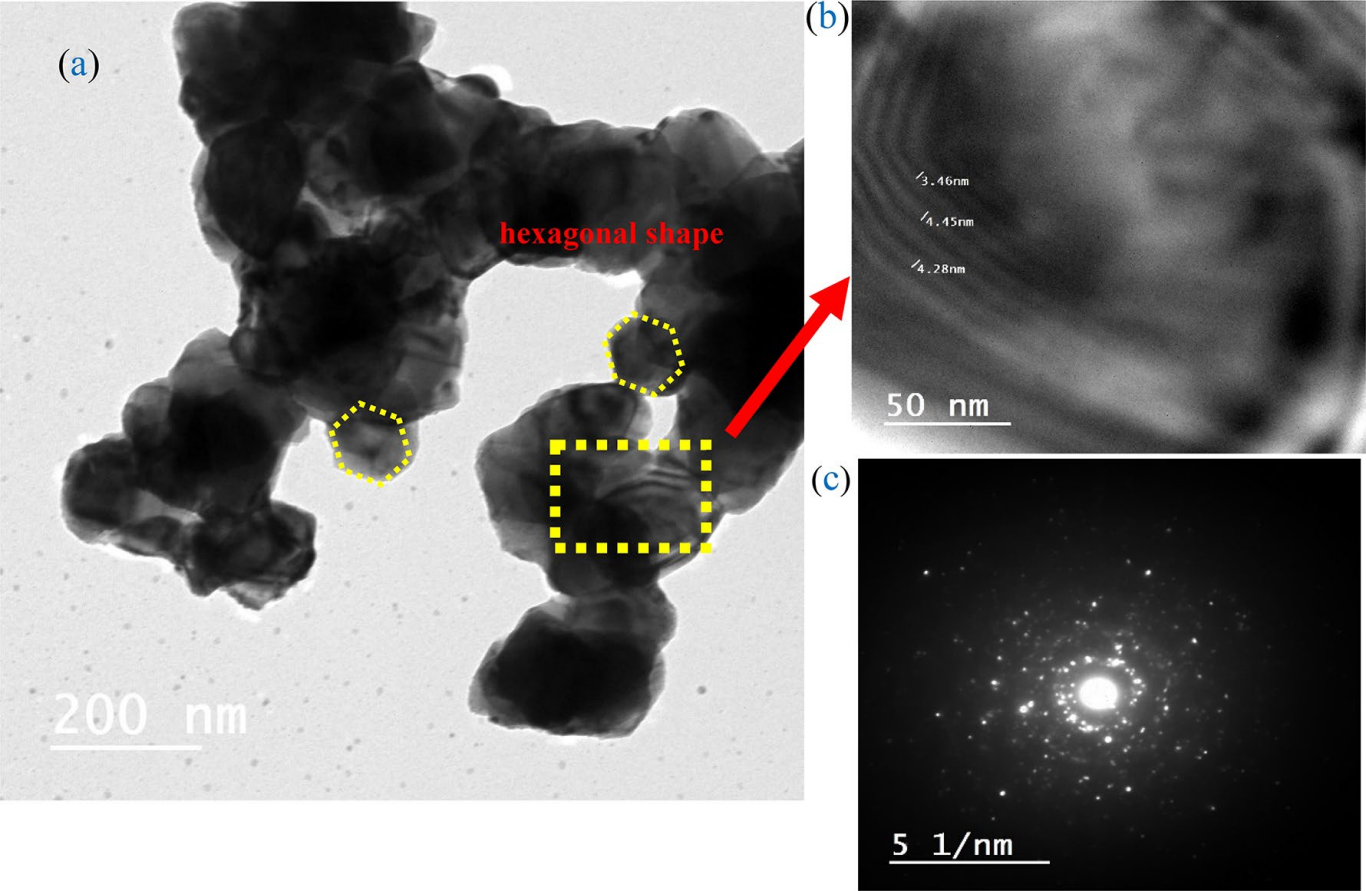
Shows (**a**) the HRTEM image, (**b**) the lattice plane image, and (**c**) the diffraction pattern for the AgCuO_2_.

Figure 3 demonstrates the HRTEM images of the typical nanocrystal. From the figure, it’s clear that the NPs are agglomerated. The obtained lattice planes correspond to the crystal plane of AgCuO_2_. The selected area electron diffraction (SAED) image verifies that the sample is in a crystalline state, which is consistent with the XRD chart.

**Figure 4:**
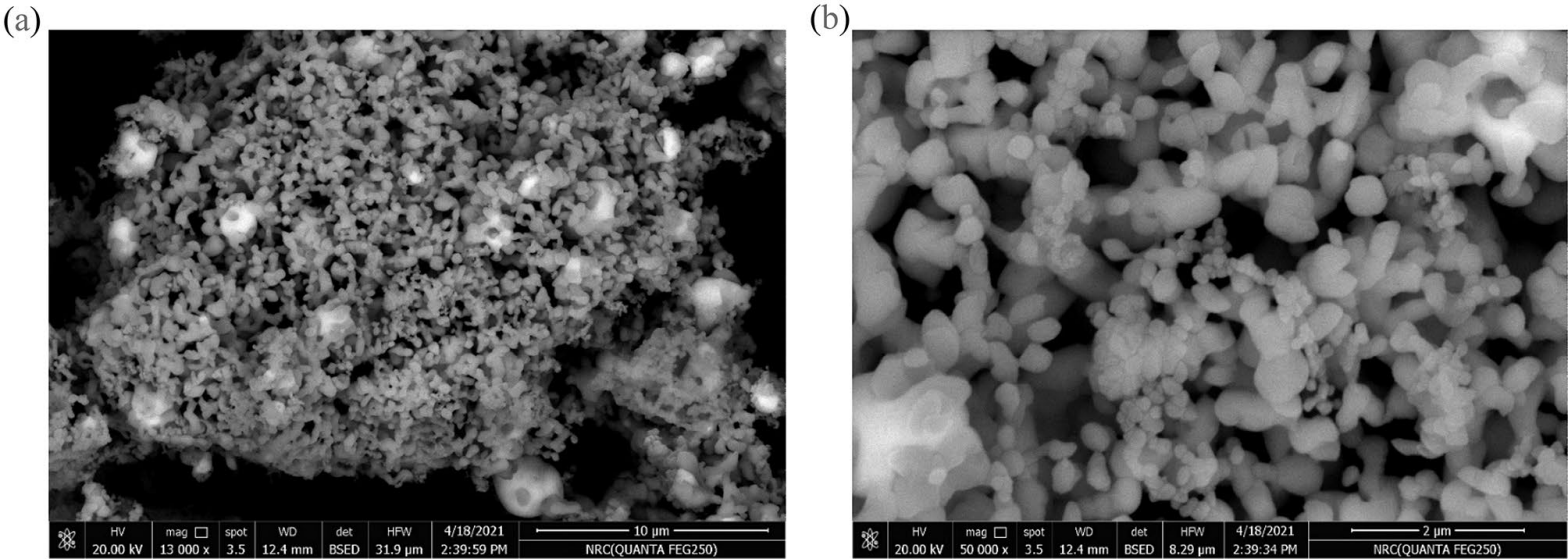
Illustrates the FESEM photographs of AgCuO_2_ at different magnifications.

Figure 4a,b illustrates FESEM photographs of AgCuO_2_. As can be seen, the AgCuO_2_ crystals are present as clusters of irregular shape. It is evident that AgCuO_2_ has a flower-like structure under high magnification, and the granular particles' surfaces are rough and made up of staked NPs. The morphology of the investigated NPs accurately depicts the hexagonal platelet-like formations with sharply defined edges, corners, and smooth surfaces.

**Figure 5:**
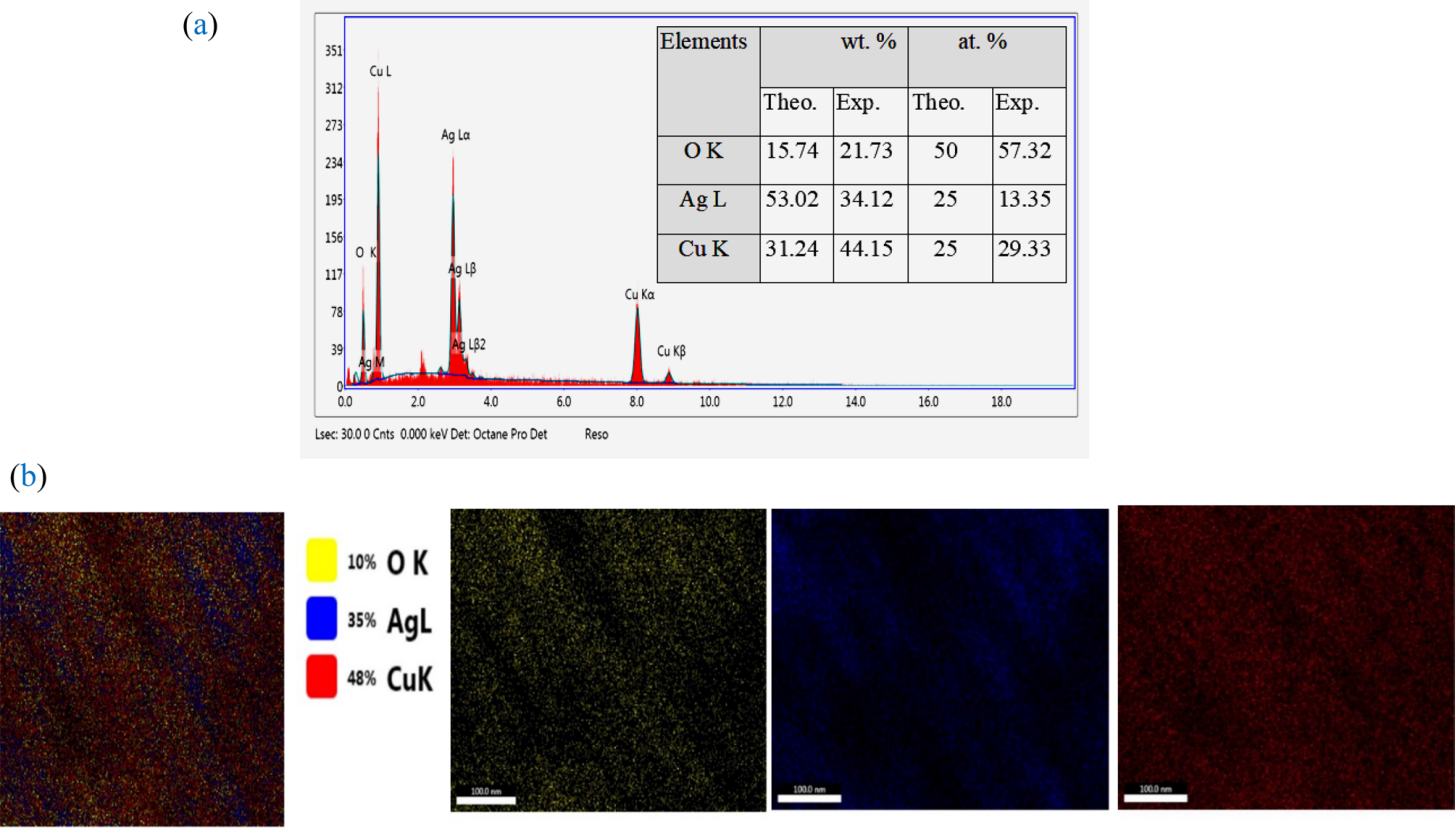
(**a**) EDAX spectra of AgCuO_2_, and (**b**) elemental mapping images.

Figure 5 illustrates the EDAX analysis of AgCuO_2_. The weight percentage (wt%) and the atomic percentage (at.%) of the elements are revealed in the inset Table. The variation between the theoretical and experimental data can be attributed to oxygen deficiency.

Figure 5b shows the elemental mapping images of Cu, Ag, and O. The images demonstrate the uniform distribution of Ag and Cu and the clustered distribution of Ag, suggesting a homogeneous structure of AgCuO_2_ NPs.

**Figure 6:**
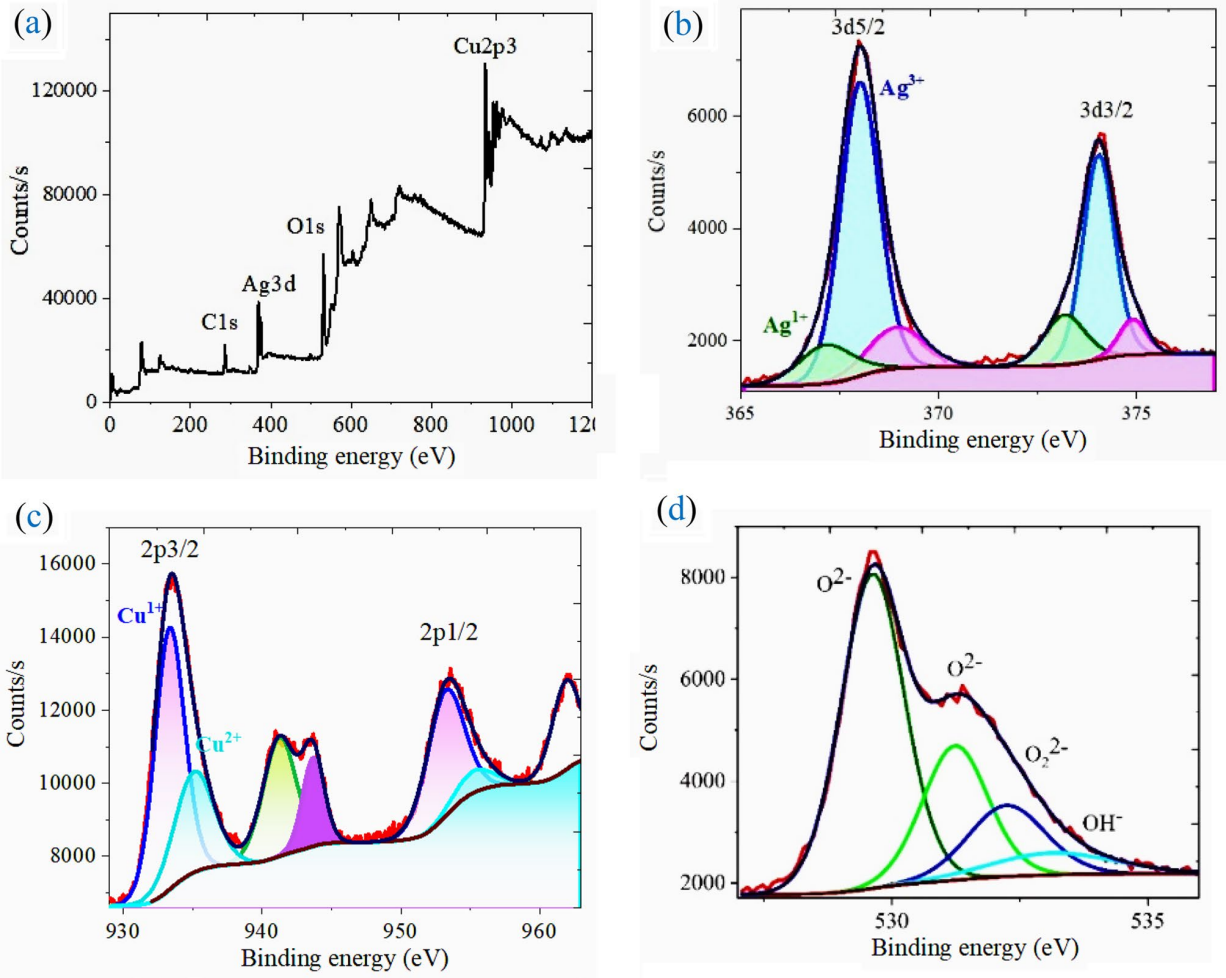
XPS spectra of (**a**) survey, (**b**) Ag 3d, (**c**) Cu2p and (**d**) O1s for the investigated sample.

The XPS survey spectrum of the sample is revealed in Fig. 6a. The existence of Cu, Ag, and O in the synthesized delafossite AgCuO_2_ NPs with no evidence of any impurity is ratified from the figure. The XPS spectrum of the Ag 3d (Fig. 6b) region is deconvoluted into Ag 3d_5/2_ and Ag 3d_3/2_ peaks at the binding energies (BE) of 367.9 and 374.08 eV, respectively. The observed doublets are attributed to either a mixture of Ag^3+^ and Ag^1+^ states on the surface of AgCuO_2_^25,26^ or to a single intermediate Ag state classified by multiple splitting^25,26^. The Ag 3d_5/2_ spectrum is deconvoluted into two peaks, indicating the presence of Ag(III) and Ag(I)^27^. The width (FWHM) of Ag 3d_5/2_ in AgCuO_2_ is 0.87 eV, which is less than that of Ag(I) oxide (FWHM 1.2 eV). This demonstrates that Ag in AgCuO_2_ has undergone more than one oxidation state^28^.

At 933.29 and 953.2 eV, respectively, the Cu 2p spectrum (Fig. 6c) can be resolved into 2p_3/2_ and 2p_1/2_^31^.The shoulder peaks located at around 935.05 and 955.27 eV, along with the satellite peaks at 943.68, 941.23, and 961.92 eV, match to Cu 2p_3/2_ and Cu 2p_1/2_ for Cu^2+^, which ratify the oxidation of Cu^29^.

The valence electron is excited to a higher energy level by an outgoing core electron_,_ which lowers the kinetic energy of the core electron, causing the appearance of the satellite peaks. Since these electrons constitute a component of the overall Cu 2p emission, it is possible to determine the ratio of Cu I: Cu II by looking at the Cu 2p_3/2_ peak and associated satellite peak areas.

Using the combined peak areas, it is anticipated that ≈ 52% of the Cu I is converted to Cu II. This estimation implies that every oxygen interstitial is coupled around a copper site, as shown in Fig. 2c.

The O 1s spectrum (Fig. 6d) can be deconvoluted into four component peaks. The oxide species (O^2−^) are accountable for the observed component peaks at 529.63 and 531.42 eV, where the peroxide species are responsible for the peak component at 532.24 eV^30,31^. At 533.13 eV^35,36^, the OH- group can be assigned to the component. One can detect a peak at 532.24 eV corresponding to peroxide species. This suggests that the excess charge on Cu (more than 1+) and Ag (more than 1+) is delocalized onto oxygen as well^28^. Moreover, based on the XPS results, the Ag/Cu ratio amounts to 1:0.99, which agrees well with the EDAX analysis.

**Figure 7:**
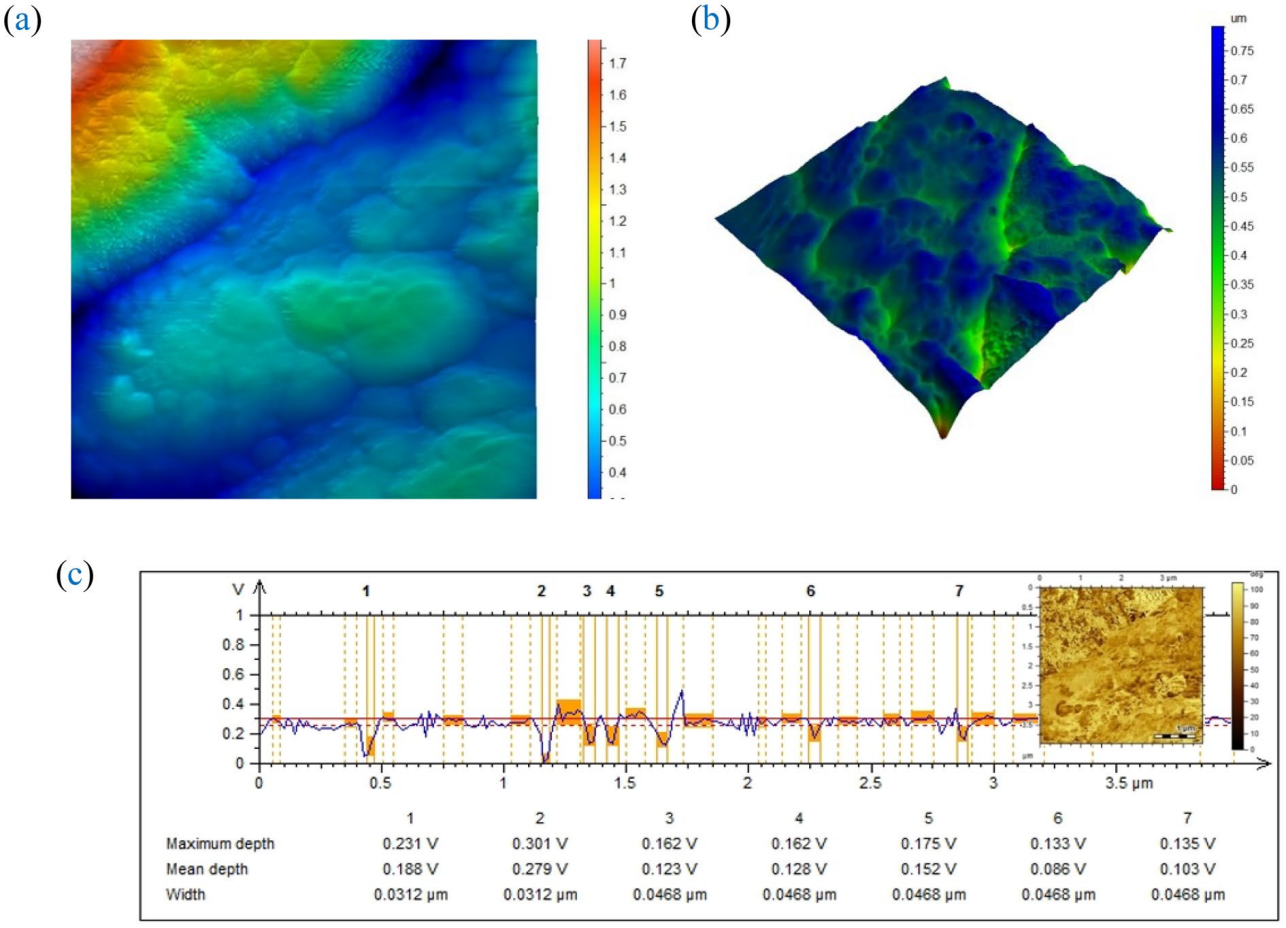
AFM micrographs for the investigated sample.

AFM investigations are carried out to characterize the surface morphologies of the AgCuO_2_ as revealed in Fig. 7a,b. The AFM images of the sample reveal grains with a triangular shape, suggesting that the AgCuO_2_ crystal's facets belong to the rhombohedral lattice. The two directions of the grains are 60° out of phase with one another.

Additionally, the line profiles of the chosen area demonstrate the change in the depth and diameter of the craters on the surface, as shown in Fig. 7c.

The roughness root-mean-square (RMS) value is 15.9 nm. The high RMS value can be attributed to the decrease in surface energy. As a result, the evolution of surface morphology follows the evolution of surface energy in a dominant fashion. The surface particles would not be spread uniformly because atoms tend to gravitate toward sites with higher energies^32–35^.

The distributed spiks are evaluated by roughness kurtosis (Rku). The roughness kurtosis has a value of 2.65 (< 3), indicating that the prepared sample is classified as bumpy surface^36^.

### Magnetic measurements

**Figure 8:**
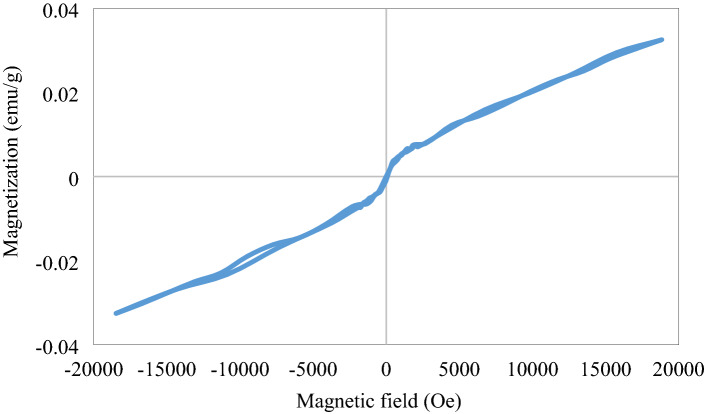
Shows the magnetic hysteresis loop for AgCuO_2_.

Figure 8 shows how the magnetization (M) depends on the applied magnetic field for AgCuO_2_. The data indicate the superparamagnetic behavior of the sample. However, the super-paramagnetic NPs exhibit a nonlinear ‘M’ plot with nearly no hysteresis or coercive field (Hc)^37^. This is in agreement with the super-magnetic behavior of nanocrystalline delafossite described by Nabiyouni et al.^38^. The magnetic parameters of the testified sample are 32.5 × 10^–3^ emu/g, 444.93 × 10^–6^ emu/g, and 41.547 Oe for the saturation magnetization (M_s_), retentivity (M_r_) and coercivity (H_c_) respectively.

The copper atom in the delafossite structure has two O_2_ atoms linearly coordinated to it, generating O–Cu–O structures that are parallel to the c-axis. This structure has the shape of a layered triangular lattice, and the O_2_ atoms in the O–Cu–O structure are all proportioned with Ag atoms that are parallel to the ab plane. The weak influence of the Ag–O–Cu interaction is owing to the larger size of Ag 4d orbitals, which increases the distance between magnetic ions and weakens interactions. The lack of hysteresis in AgCuO_2_ signifies that long-range ordering is not present.

### Antimicrobial activity (AMA) of AgCuO_2_

The as-synthesized AgCuO_2_ is assessed for in-vitro AMA by the disc diffusion protocol. *Bacillus subtilis (B. subtilis), Bacillus cereus (B. cereus), Enterococcus faecalis (E. faecalis),* and *Staphylococcus aureus (S. aureus)* are used as model gram-positive bacteria in this study. While *Escherichia coli (E. coli), Neisseria gonorrhoeae (N. gonorrhoeae), Pseudomonas aeruginosa (P. aeruginosa),* and *Salmonella typhimrium (S. typhimrium),* are model gram-negative bacterium^39–41^. *Candida albicans (C. albicans),* and *Aspergillus flavus (A. flavus)* are model fungi.

**Figure 9:**
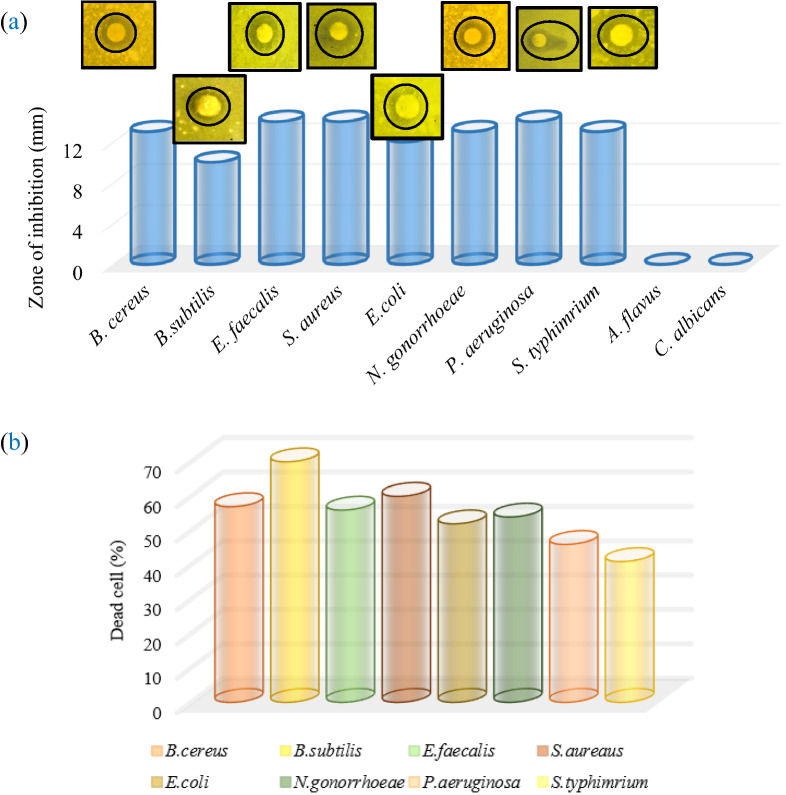
(**a**) The bar values for the achieved zones of inhibition with an in-site image for the inhabitation zone and (**b**) the dead cell %.

Figure 9a presents the bar values for the achieved zones of inhibition (ZI) with an in-site image for the ZI. According to the results, the AgCuO_2_ sample has a relatively high ZI against both bacterial strains (+ and −), but no activity against fungus.

AgCuO_2_ plays a pivotal role that accounts for its effective bactericidal properties. AgCuO_2_ shows promise in terms of stability, ease of recovery, and low toxicity. Applying AgCuO_2_ also limits the aggregation and leaching of metallic silver ions. The proven chemical mode of action for inactivating microorganisms is oxidative stress, which is brought on via charge transfer and in-situ reactive oxygen species^42^.

The AMA of AgCuO_2_ is derived from the following three mechanisms. According to the first mechanism, Ag^+^ acts at the membrane level because it can pass through the outer membrane and accumulate in the inner membrane, where its adhesion causes damage and destabilization, leading to increased membrane permeability and, ultimately, cell death^43,44^.

In the second mechanism, the negative charges on the bacteria's surface, caused by the carboxyl and phosphoric acid groups in the outer membrane, cause Ag^+^ to easily concentrate around the active bacterial cells^45^. The thiol (-SH) group of the cysteine chain might interact with the adsorbed Ag^+^ by substituting the hydrogen atom to generate –S–Ag, which impairs the affected protein's ability to perform its enzymatic function and prevents the growth of bacterial cells.

On the other hand, the third mechanism suggested that Ag^+^ releases from the NPs and interacts with cellular components, changing metabolic pathways, membranes, and even genetic material. The third mechanism can be proposed to happen concurrently with the other two^46,47^.

The percent of viable cells is calculated using the following equation^48^:

1 $$\mathrm{Dead \; cell \; Percent }\left(\mathrm{\%}\right)=\frac{diameter \; of \; control -diameter \; of \; treated \; sample }{diameter \; of \; control }\times 100$$

The efficiency of the killing of bacteria is presented in Fig. 9b. The maximum efficiency is obtained for *B. subtilis* (70%), while *S. typhimrium* shows the minimum one (41%). The differences in cell wall structure of bacteria play an important role in antimicrobial susceptibility, in this regard, the sample has different activity against Gram-positive and Gram-negative bacteria^49,50^.

The multi-oxidation states (Cu (I, II) and Ag (I, III)), and the presence of ROS as relevant from XPS results, besides the nanometric-size, assure the antimicrobial activity of the prepared sample.

Finally, one can conclude that the excellent bactericidal performance of AgCuO_2_ has been revealed. Thus, it can be used in biomedical and wastewater disinfection applications. Further analysis to confirm the efficiency of AgCuO_2_ will be performed using other experiments in the future.

## Conclusion

The AgCuO_2_ NPs were successfully synthesized via an easy, low-cost technique. The size and shape of AgCuO_2_ NPs, along with their structural polytypes of 2H (hexagonal) or 3R (rhombohedral), are dependent on the preparation conditions. The crystallite size (D) and the ε are found to be 36.23 nm and 1.02946 × 10^–4^ respectively, demonstrating a comparatively low lattice strain in AgCuO_2_. The hexagonal platelet-like structures with clearly defined edges, corners, and smooth surfaces are well represented by the morphology of the produced particles. The magnetic parameters are recognized, and the superparamagnetic property is achieved at room temperature. AgCuO_2_, which combines magnetic and highly AMA properties, provides easier separation and high efficiency. As a result, the mineral CuAgO_2_ is used in medicine as a novel antimicrobial substance.

## Data Availability

The authors declare that all the data supporting the findings of this study are available in the Crystallography Open Database (COD) repository [entries; 3000419 and 3000420].
